# Connecticut Valley Dental Society

**Published:** 1892-08

**Authors:** 


### CONNECTICUT VALLEY DENTAL SOCIETY.

### DISCUSSION OF DR. BUCK'S PAPER ON GENERAL ANÆSTHESIA.

(For Dr. Buck's paper, see page 578.)

*Dr. Rider.*—I would like to ask Dr. Buck, as he has spoken of the low temperature, is it not a favorable symptom? Also, what is his authority for saying that oxygen is lost from the nitrous oxide in its administration?

*Dr. Buck.*—My answer to the first question is, I think it is; and to the second, I do not remember. My experience is that we have the same symptoms with nitrous oxide as with carbonic acid gas. One is as poisonous as the other. Of the two, I prefer to give ether.

*Dr. Lovejoy.*—This is an exhaustive subject, and the only way to properly discuss it is to have copies of the paper put into the hands of able men before the meeting. No one anæsthetic is the best in all cases. There is a great deal said against chloroform, but it is very satisfactory in some places; a few drops combined with the nitrous oxide is of great advantage. I know some surgeons will not have anything to do with it, but in Germany and the South it is preferable to other anæsthetics. I administer it when it is about the only thing that can be used. Children sometimes will not take the gas, and then chloroform can be used. It acts quickly. This is a subject of great interest to dentists.

*Dr. Rider.*—One thing to be said in favor of nitrous oxide is, it is the safest anæsthetic in the hands of the incompetent: in their ignorance the symptoms become so alarming before the actual danger-point is reached, that it frightens them and saves the patient. I think this is the reason there has been so little trouble with it in the hands of the ignorant practitioner.

*Dr. Morgan.*—One word that has been dropped I wish to speak of, and that is death. In the discussion of anæsthetics and its attendant symptoms, we are constantly reminded of this one supreme danger, the death of a patient in our chair. I would like to inquire if we do not mistake in this matter. It is certainly a serious thing to have a death in our office; but so far as the patient is concerned, is it not possible that it is preferable to the continued life with the train of evils induced by the anæsthetic,—practically a living death? I make this mention because I feel that health should be as sacred to us in these cases as life.

*Dr. McManus.*—Years ago I saw chloroform and ether administered a great many times in the most simple way. I never saw a bad case. It is very unfortunate that there is so much conflict of opinion among surgeons. Every one admits chloroform has more deaths than other anæsthetics; ether, next to gas, the least of all. The point Dr. Morgan makes is a good one. The deaths from chloroform have mainly occurred in hospitals and upon cases when not in good condition. Many occur before any operation has been performed. This seems to show that the operator should know his patient before administering anything. Patients attribute ill results to the anæsthetic when it may not be that at all. A surgeon generally prepares his patient before giving the anæsthetic; dentists do not.

*Dr. Maxfield.*—There is much weight to the remarks of Dr. Morgan. While it is an admitted fact that at the time of administration there are more deaths when chloroform is used than when ether, yet, if we could know all the lesions that are the result from ether, such as renal troubles, etc., I am inclined to think we could class ether as dangerous as chloroform. I believe that, in the near future, pathologists will be able to throw much light on this subject. We must admit that nitrous oxide is the safest of all anæsthetics. Its extensive use by so many unprincipled and ignorant men, and the few accidents that occur, demonstrate this. Undoubtedly a great many after-troubles can be traced back to the gas, as the inciting cause, for I do not believe the normal functions can be so interrupted, as they are when an anæsthetic is administered, without causing lesions. One thing we should always bear in mind, whenever we are administering an anæsthetic, and that is we are carrying the patient as near death as possible, yet keep him alive. It is a great risk, and one I do not like to take. I always feel a relief when the patient "comes to," whether it is gas or ether I have given.

*Dr. F. E. Nims.*—I do not believe in the use of paper cones, teacups, and like appliances to force the patient in taking ether, for it frightens the patient and induces strangling, coughing, etc. I prefer to use a towel folded as it comes from the laundry, and placing the upper part close to the face under the eyes, and then pouring on small quantities of ether at short intervals, doing this as the patient exhales. I do not feel, as some who have spoken, that danger does not exist when using nitrous oxide. I had a case not long ago, of syncope after extraction with nitrous oxide, which, to say the least, was very unpleasant. Only a few weeks ago a man died while sitting in a barber's chair in Fitchburg. Had he come to me and I had given him nitrous oxide, it would have had the credit of causing his death. We do not know what conditions patients may be in when they come to us and request us to administer an anæsthetic.

*Dr. A. J. Nims.*—After an experience of over fifteen years in the use of ether and nitrous oxide, and from a close study of the experiments and investigations of those who are the best authority on these agents, I have reached the conclusion that ether is as safe as nitrous oxide. The administrator should have a technical knowledge, experience, and good common sense. Many unpleasant experiences with ether are caused by the bungling manner in which it is given. Many physicians exhibit poor judgment in handling it. The strong point with ether is that by observing the symptoms, serious trouble may be averted. In the use of nitrous oxide some men attempt extensive operations and administer too much gas, and thus almost produce asphyxia, and also, perhaps, having a little chloroform introduced into the vapor. It is in such cases as these you get those living deaths spoken of by Dr. Morgan. I have talked with a number of people who have taken gas under these conditions, and they have remarked that nothing would induce them to take gas again in those offices.

*Dr. McManus.*—In talking with Dr. Atkinson some years ago he made a remark that he thought a great deal of the nervous trouble here met with was the result of administering an anæsthetic to the mother at time of childbirth. We know that the majority of nervous troubles occur among the well-to-do people, those who can afford to have every attention. Among the poor they have to get along without this luxury.

*Dr. Maxfield.*—It is a fact that with children and with women in time of labor, chloroform acts differently; that is, it is as safe as any anæsthetic. What Dr. McManus has just remarked is being recognized by the more intelligent physicians, and anæsthetics are not given at time of childbirth as often as a few years ago. Only a little chloroform or ether is used, just enough to cause a slight benumbing effect, not enough to put the mother to sleep or to approach unconsciousness. Only a short time ago a physician remarked in regard to two of my patients,—a brother and sister,—that he thought the cause of their being so nervous was the result of the anæsthetic used at the time of their birth.

